# Transcutaneous Electrical Acupoint Stimulation Improved Preoperative Blood Pressure in Gynecological Malignant Tumor Patients With Hypertension: A Randomized, Controlled Trial

**DOI:** 10.3389/fonc.2022.906528

**Published:** 2022-06-01

**Authors:** Liang Chen, Yang Shen, Shuangmei Liu, Yanyan Cao

**Affiliations:** ^1^Department of Anesthesiology, Shengjing Hospital of China Medical University, Shenyang, China; ^2^Department of Emergency Medicine, Shengjing Hospital of China Medical University, Shenyang, China

**Keywords:** malignant tumor, hypertension, preoperative, electrical stimulation, acupoint, complementary therapy

## Abstract

**Objective:**

Gynecological malignant tumor patients with hypertension, even if blood pressure is well controlled, are prone to hypertension before surgery. We plan to verify the effect of transcutaneous electrical acupoint stimulation (TEAS) on stabilizing blood pressure before operation.

**Methods:**

We enrolled 91 patients and randomly divided them into TEAS group (n=46) and control group (n=45). Patients in TEAS group received TEAS at acupoints Hegu and Neiguan. Patients in control group received transcutaneous electrical stimulation at the nonacupoint position of the upper limbs. After entering the operating room, the blood pressure before and after induction was measured. The main results were the occurrence of preinduction hypertension and postinduction hypotension.

**Results:**

There was no difference in the general information of the two groups. There were four cases (9%) of preinduction hypertension in TEAS group and 13 cases (29%) in control group. The incidence in TEAS group was significantly lower (P=0.013). There were five cases (11%) of postinduction hypotension in TEAS group and eight cases (18%) in control group. There was no significant difference between the two groups (P=0.346). The systolic blood pressure (SBP), diastolic blood pressure (DBP) and mean blood pressure (MBP) of the highest blood pressure before induction in TEAS group were lower than those in control group (P=0.002, 0.002, and 0.001). There was no difference in SBP, DBP, or MBP between the two groups on the day before the operation. There was no difference in the lowest blood pressure before operation between the two groups after induction

**Conclusion:**

TEAS can prevent preinduction hypertension in patients with gynecological malignant tumors.

**Clinical Trial Registration:**

http://www.chictr.org.cn/showproj.aspx?proj=143276, identifier ChiCTR2100054336.

## 1 ntroduction

Perioperative hemodynamic stability is very important for the rehabilitation of patients with malignant tumors, but it is not easy to maintain blood pressure stability. Especially for gynecological malignant tumor patients with essential hypertension, even if they use drugs regularly to control blood pressure, there are often obvious blood pressure fluctuations in the perioperative period. These blood flow fluctuations will aggravate the damage to target organs, affect the perfusion of important organs and increase perioperative cardiovascular risk. It may also delay surgery and increase hospital stay and costs.

The most common changes in preoperative blood pressure include pre-induction hypertension and postinduction hypotension. Pre-induction hypertension is generally considered to be related to tension and increased sympathetic excitability. Preoperative general use of midazolam or increased use of antihypertensive drugs can reduce the incidence of preinduction hypertension. Midazolam may affect consciousness and breathing, which is dangerous for elderly and critically ill patients. If patients add antihypertensive drugs before surgery, it may lead to hypotension. Postinduction hypotension easily occurs in patients with malignant tumors and is generally considered to be related to cardiovascular inhibition caused by narcotic drugs. Clinically, the method of reducing the dose of anesthesia-inducing drugs is often used to prevent hypotension, but there is no clear guide for the degree of drug dose reduction. Previous studies have shown that transcutaneous electrical acupoint stimulation (TEAS) can stabilize blood pressure during anesthesia awakening and extubation (1–3). However, the effect of TEAS on preoperative blood pressure has not been studied.

## 2 Methods

### 2.1 Study Design

We conducted a prospective, single-blind, parallel randomized, controlled trial. This study was approved by the Medical Ethics Committee of Shengjing Hospital of China Medical University (approval number: 2021PS127J, approval date: April 25, 2021). The trial was registered at the Chinese Clinical Trial Registry (registration number: ChiCTR2100054336, registration date: December 14, 2021). Patients were recruited after trial registration. The patients were randomly divided into TEAS group and control group. All patients provided written informed consent before participating in the study. This study complied with the Declaration of Helsinki.

### 2.2 Study Subjects

Inclusion criteria: Patients with gynecological malignant tumors and who were diagnosed with essential hypertension before or after hospital admission. Hypertension was defined as systolic blood pressure (SBP) ≥140 mmHg and/or diastolic blood pressure (DBP) ≥90 mmHg in blood pressure measurements on three different days. American Society of Anesthesiologists (ASA) grade I-II.

Exclusion criteria: Patients with secondary hypertension; skin damage, rash, or local infection at sites to be used for electrical stimulation or the presence of metal implants near the sites; implantation of pacemakers, cardiopulmonary pacemakers, or defibrillators; implantation of electronic insulin pumps or other types of electronic implanted devices; who were pregnant or lactating; body mass index (BMI) ≥30 kg/m2; central nervous system disorders or mental illness; or who had been treated with electrical stimulation within a week.

### 2.3 Intervention

The treatment plan for hypertension in this study was in accordance with the “consensus of anesthesia management experts for perioperative hypertensive patients” formulated by the Chinese Society of Anesthesiology (4). The patients’ blood pressure was measured under identical conditions using the same model monitor and cuff of appropriate size. The patients were asked to recline in a supine position and rest comfortably for 5 min before measurement of the blood pressure in their left upper extremity. The blood pressure measurement was performed three times at 3-min intervals, and a mean value was obtained. The blood pressure of all patients was required to be < 140/90 mmHg for more than 3 days.

The patients were subjected to transcutaneous electrical stimulation to the lateral sides of both forearms using the Genuine Hwato SDZ-II electronic acupuncture therapy instrument. The stimulation time was 5 minutes, and the intensity was 5 mA to ensure that patients can always receive percutaneous electrical stimulation in subsequent trials.

One hour before surgery, the patients were intravenously given 0.05 mg/kg midazolam, with a total dose of <5 mg administered. An investigator performed the corresponding interventions according to the grouping of the patients. Electrical stimulation was performed alternately every six seconds at 2 Hz and 10 Hz using the Genuine Hwato SDZ-II electronic acupuncture therapy instrument.

**Figure 1:**
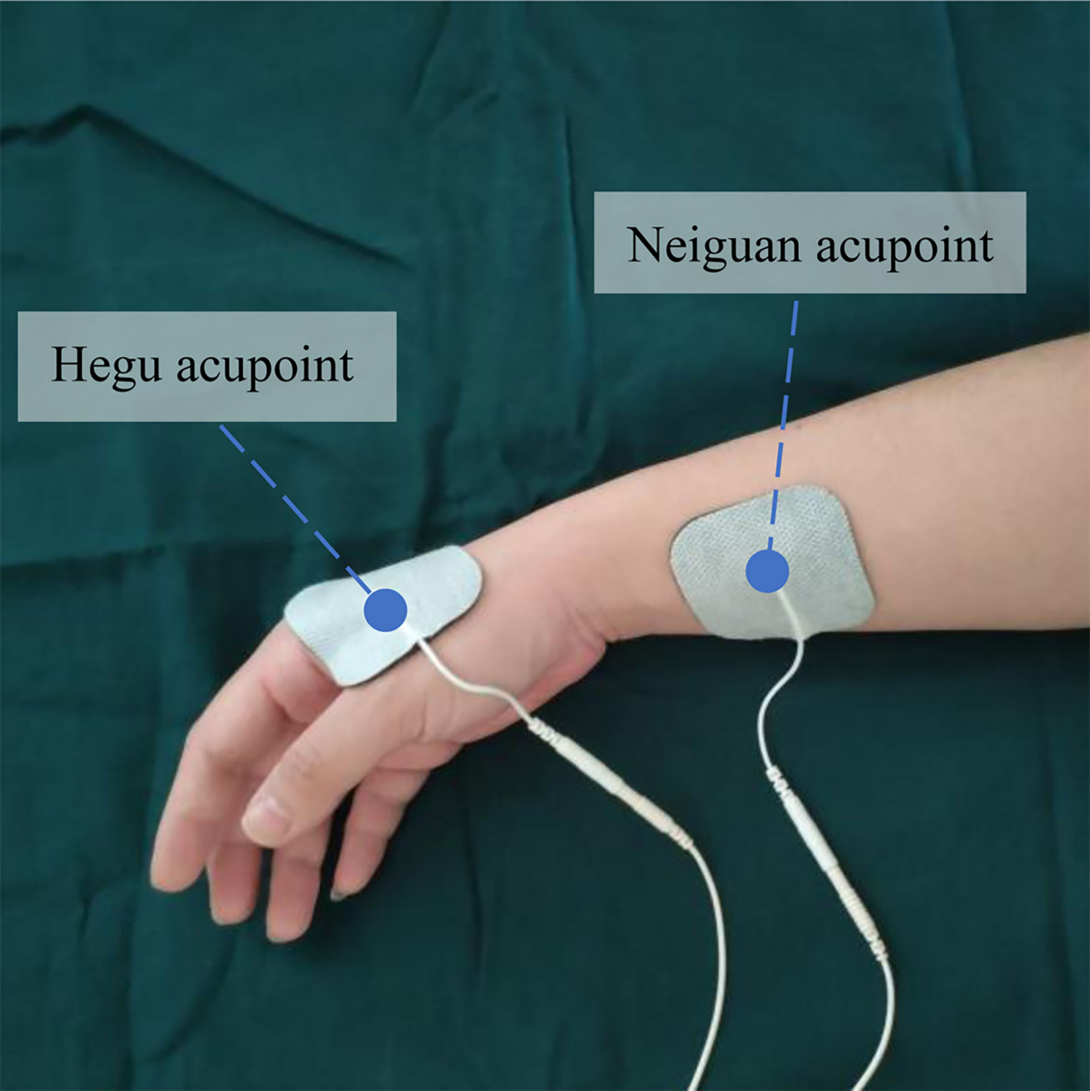
Location diagram of the Hegu and Neiguan acupoints.

In TEAS group, the patients were administered acupoints Hegu (LI4) and Neiguan (PC6) on both sides of the forearms through the pasted electrode, with an intensity of 10 to 15 mA. The Hegu acupoint is located between the first and second metacarpal bones at the midpoint of the radial side of the second metacarpal bone. The Neiguan acupoint is located on the palm side of the forearm and 2 inches below the wrist between the palmaris longus tendon and the flexor carpi radialis tendon, as shown in **Figure 1**. The intensity is set as the intensity at which the patient has a strong tremor but no obvious tingling. During electrical stimulation, the patient could request modifications to the intensity of the electrical stimulation.

In control group, the patients were provided transcutaneous electrical stimulation on the non-acupoint area on the dorsal side of the forearms through pasted electrodes with an intensity of 5 mA. Patients could ask for a reduction in the intensity of stimulation.

After entering the operating room, the patient was supine on the operating table and covered with a lightweight blanket. ECG and pulse oxygen saturation were monitored. The patient inhaled oxygen at a flow rate of 3 L/min through a nasal catheter. After the patient rested for 5 mins, blood pressure was measured in the left upper limb.

If the patient had an SBP >180 mmHg or a DBP >110 mmHg, A 10-mg urapidil injection was administered intravenously. Blood pressure was measured again after three minutes. If the SBP was still >180 mmHg or DPB >110 mmHg, urapidil was repeated. If the dose of urapidil reached 50 mg, the systolic blood pressure was still > 180 mmHg or the diastolic blood pressure was still > 110 mmHg, the surgery would be canceled.

Anesthesia induction was performed in patients with qualified blood pressure. After induction, the patients’ blood pressure was measured again. When the blood pressure drops more than 30% of the blood pressure before induction, vasopressor drugs are used. The electrical stimulation was stopped before the operation, and the electrodes and electrical stimulation devices were withdrawn.

### 2.4 Outcome Measurements

#### 2.4.1 Primary Outcomes

1. Pre-induction hypertension: In the operating room, before using antihypertensive drugs, patients with SBP > 140 mmHg or DBP > 90 mmHg were defined as having preinduction hypertension.
2. Postinduction hypotension: After induction, before surgery, the mean blood pressure (MBP) was lower than 30% before induction, which was defined as postinduction hypotension. MBP=(SBP+2DBP)/3.

### 2.5 Secondary Outcomes

1. Blood pressure: T1 blood pressure, blood pressure measured the day before operation; T2 blood pressure, highest blood pressure before induction; T3 blood pressure, the lowest blood pressure before operation after induction.
2. The final intensity before the end of the electrical stimulation and the duration of electrical stimulation.
3. Adverse reactions such as redness, swelling, or itching of the patient’s skin were monitored and recorded within 24 hours after electrical stimulation.

### 2.6 Statistical analysis

#### 2.6.1 Randomization and Blinding

The computer-generated random allocation sequence was created by an independent investigator using SPSS with a 1:1 allocation and random block size. The participants were also recruited by this investigator. The participants were randomized using sealed opaque envelopes to reveal the treatment arm on the morning of surgery.

The participants were blind to grouping and could not judge grouping from electrical stimulation. The investigators responsible for the interventions used gauze to block the participants’ arms and stimulator. The investigators who performed anesthesia induction, and follow-up were blind to the grouping.

### 2.7 Sample Size Calculations

Based on the results of the pre-test, we ascertained that the probability of preoperative hypertension in hypertensive patients was 30%, and the intervention was expected to reduce the incidence by half to 15%. To achieve 90% statistical power (β=0.1) with a two-sided confidence interval of 95% (α=0.05), we used PASS11 to calculate that 39 patients should be recruited to each group. The study plans to recruit 20% more patients, i.e., up to 46 patients in each group, to account for potential loss to follow-up and withdrawals.

### 2.8 Statistical Methods

All data were based on the intention-to-treatment (ITT) population, which was defined as all patients who received electrical stimulation, regardless of the duration of electrical stimulation. For patients who stopped the operation for any reason, when they entered the operating room, their blood pressure was measured and included in the statistics as the blood pressure before anesthesia induction. Even if the patient was not anesthetized, it was used as one of the sample sizes for blood pressure statistics after induction.

We used SPSS 24.0 software (IBM, Armonk, NY) for data processing and statistical analysis in this study. Continuously distributed variables are presented as the mean ± standard deviation (□x± sd) or median (interquartile range). The independent-sample t-test and the Wilcoxon rank-sum test were used to compare the two groups. The counting data are presented as the number of cases (percentage, 95% confidence interval [CI]), and we used the chi-squared test or Fisher’s exact probability test for their analysis. P < 0.05 was considered a statistically significant difference.

## 3 Results

**Figure 2:**
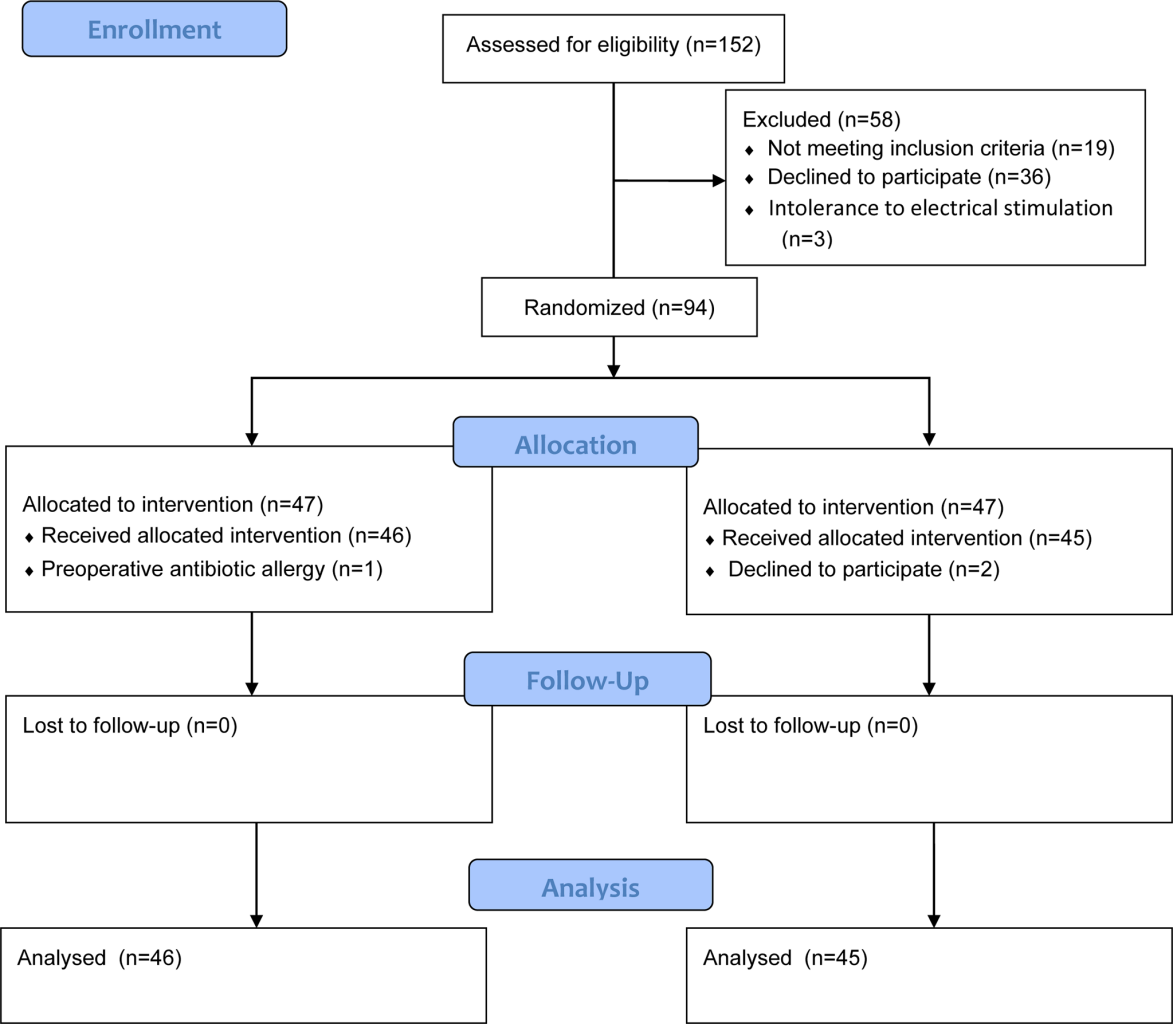
Consolidated Standards of Reporting Trials (CONSORT) flow diagram.

This study was conducted in Shengjing Hospital of China Medical University from December 2021 to March 2022. A total of 152 patients were recruited, and 91 patients completed the study. In control group, two patients canceled the operation because of arrhythmia before induction, and the other 89 patients underwent anesthesia as planned. The consort flow chart is shown in **Figure 2**. **Table 1** shows the general information of the two groups, including demographic data such as age, height, weight, and body mass index, types of antihypertensive drugs used before the operation, and types of surgery. There was no significant difference between the two groups.

**Table 1:** General information for both groups.

	TEAS group (n=46)	Control group (n=45)	Statistical value	*P* value
Age (years)	57.9 ± 8.6	56.8 ± 9.3	0.594	0.554
Height (cm)	162.9 ± 3.3	162.5 ± 3.3	0.455	0.651
Weight (kg)	61.9 ± 4.8	64.1 ± 5.6	-1.981	0.051
BMI (kg/m^2^)	23.6 ± 1.9	23.9 ± 2.3	-0.647	0.519
Concurrent diabetes	4 (8.7%)	3 (6.7%)	0.132	1.000
Types of antihypertensive drugs			2.043	0.728
1 β-blockers 2 Calcium channel blockers 3 ACEI/ARB 4. diuretic 5. Combination or compound preparation	2 (4%) 24 (52%) 15 (33%) 1 (2%) 4 (9%)	3 (7%) 22 (49%) 11 (24%) 2 (4%) 7 (16%)		
Types of surgery			0.320	0.852
1 Nonabdominal incision surgery 2 Transabdominal incision surgery 3 Laparoscopic surgery	17 (37%) 25 (54%) 4 (9%)	18 (40%) 22 (49%) 5 (11%)		

Data are presented as the mean ± standard deviation or number (%).

BMI, Body Mass Index.

There were four cases (9%) of preinduction hypertension in TEAS group and 13 cases (29%) in control group. The incidence of preinduction hypertension in TEAS group was lower than that in control group, and the difference was statistically significant (statistical value 6.106, P=0.013).

There were 5 cases (11%) of postinduction hypotension in TEAS group and 8 cases (18%) in control group. There was no significant difference between the two groups (statistical value 0.887, P=0.346).

There was no difference in SBP, DBP, or MBP at T1 between the two groups. At T2, SBP, DBP, and MBP in TEAS group were lower than those in control group, and the difference was statistically significant (P=0.002, 0.002, and 0.001). There was no difference in SBP, DBP, or MBP at T3 between the two groups. The blood pressure at each time point is shown in **Table 2**.

**Table 2:** The blood pressure at each time point.

	TEAS group (n=46)	Control group (n=45)	Statistical value	*P* value
T1				
SBP DBP MBP	122.8 ± 4.8 73.3 ± 5.4 106.2 ± 4.0	123.2 ± 6.4 74.5 ± 7.4 107.0 ± 5.8	-0.372 -0.838 -0.753	0.711 0.404 0.454
T2				
SBP DBP MBP	126.7 ± 6.2 77.5 ± 6.5 110.3 ± 5.4	132.9 ± 11.7 82.5 ± 8.0 116.1 ± 9.8	-3.173 -3.252 -3.474	0.002 0.002 0.001
T3				
SBP DBP MBP	105.2 ± 8.9 60.6 ± 10.8 90.4 ± 8.1	102.6 ± 9.0 62.6 ± 9.4 89.3 ± 7.619	1.378 -0.959 0.638	0.172 0.340 0.525

Data are presented as the mean ± standard deviation.

T1, blood pressure measured the day before operation; T2, highest blood pressure before induction; T3, the lowest blood pressure before operation after induction.

SBP, Systolic blood pressure; DBP, diastolic blood pressure; MBP, Mean blood pressure.

There was a difference in the intensity of electrical stimulation between the two groups but no difference in the time of electrical stimulation. Although two patients in TEAS group and one patient in control group manifested redness, swelling, and itching at the TEAS site, these symptoms were alleviated within six hours in both groups of patients, with no significant differences between them, as shown in **Table 3**.

**Table 3:** Electrical stimulation intensity and duration and adverse reactions in the two groups.

	TEAS group (n=46)	Control group (n=45)	Statistical value	*P* value
Electrical stimulation intensity (mA)	13 (2)	5 (0)	0.000	<0.001
Duration of electrical stimulation (min)	64 (5)	67 (3)	811.500	0.074
adverse reaction	2 (4%)	1 (2%)	0.322	1.000

Data are presented as the median [interquartile range] or number (%).

## 4 Discussion

This study found that compared with control group, the incidence of preinduction hypertension and the average value of blood pressure before induction in TEAS group were lower. There was no difference in the incidence of postinduction hypotension or the average value of blood pressure after induction between the two groups.

TEAS is a non-invasive electrical stimulation technology that has the advantages of safety, simplicity, and ease of use (5, 6). TEAS uses pulse current to generate electrical stimulation through the connecting electrode corresponding to acupoints on the skin, which combines the effects of transcutaneous electrical nerve stimulation and acupoint stimulation (7–10). There are some differences between TEAS and acupoint stimulation, such as electroacupuncture and acupuncture. Electroacupuncture and acupuncture directly act on acupoints at the exact location, while TEAS uses electrodes to act on the skin including acupoints.

Previous studies have shown that TEAS can effectively reduce the dosage of opioids and stress response during extubation (11–13). In a study of craniotomy in elderly patients, it was found that TEAS could reduce the stress response during extubation, and the hemodynamic parameters and plasma concentrations of epinephrine, norepinephrine, and cortisol in TEAS group decreased during extubation (2). Huang et al. applied TEAS to patients with video-assisted thoracoscopic lobectomy 30 minutes before induction and during surgery. TEAS reduced intraoperative opioid consumption, extubation time, and postoperative pain score (3).

The mechanism of TEAS may play an analgesic role by activating endogenous pathways. The sensory nerve endings activated by electrical stimulation are transmitted to the spinal cord and brain, which can not only directly inhibit spinal cord interneurons sensitive to opioids but also trigger the release of endogenous opioids, such as endorphins, enkephalins, and Dynorphins (14, 15).

Different frequencies of electrical stimulation can activate different endogenous opioids. Low-frequency electrical stimulation mainly leads to the release of met-enkephalin (M-ENK) and β-endorphin (β-EP); medium-frequency stimulation releases M-ENK and dynorphin-A (DYN-A), and high-frequency stimulation mainly leads to the release of DYN-A in the spinal cord (16–18). The effect of low-frequency stimulation on pain often lasts longer than electrical stimulation itself, while the effect of high-frequency stimulation disappears soon after stopping stimulation (16–18).

Clinical practice suggests that alternating stimulation with low and high frequencies can also induce a more potent analgesic effect (19). A study showed that the use of 2 Hz and 100 Hz alternating stimulation can induce the release of M-ENK, β-EP, and DYN-A, and its effect was stronger than that of 2 Hz or 100   Hz alone (20). In our study, medium- and low-frequency electrical stimulation was used, with two different frequencies of 2 Hz and 10 Hz alternating for 6 seconds. When electrical stimulation with different frequencies is used alternately, the corresponding endogenous opioid peptides can be released at the same time to exert an overall synergistic analgesic effect. Alternative stimulation with different frequencies can also avoid tolerance and achieve a better stimulation effect.

In addition to activating endogenous opioid peptides, TEAS can also inhibit hippocampal β Wave and activation δ Wave to regulate EEG activity and neurotransmitter production in the brain to effectively reduce neural pressure and trigger sedation and hypnosis in patients (21).

Previous studies have taken TEAS as a direct treatment for hypertension and achieved curative effects (22–25). The mechanism of the hypotensive effect of electrical stimulation is unclear. A study showed that electrical stimulation of Neiguan (PC6) can activate the C fiber of the median nerve to alleviate hypertension (26). Animal experiments have shown that acupuncture plays an antihypertensive role by improving oxidative stress and the redox-sensitive pathway in the ventrolateral medulla oblongata of spontaneously hypertensive rats (27). Electrical stimulation can also reduce the activity of the sympathetic nervous system and increase the activity of the parasympathetic nervous system in patients with hypertension (28). In these studies, it takes a long time for electrical stimulation to produce antihypertensive effects. Compared with the time of single TEAS before surgery, the above reasons may not be enough to directly reduce blood pressure before induction.

After induction, there was no difference in blood pressure between the two groups, indicating that TEAS will not increase the risk of hypotension after induction while reducing preoperative hypertension. The reason may be that the analgesic and sedative effects of TEAS are smaller than those of anesthesia-inducing drugs and will not increase the effects of these drugs.

In the experiment, we selected Hegu (LI4) and Neiguan (PC6) for electrical stimulation. According to the theory of traditional Chinese medicine, Hegu (LI4) and Neiguan (PC6) are important acupoints for analgesia, which have been confirmed in various animal models (29, 30). Moreover, these two acupoints are located in the upper limb, are convenient and accurate on the body surface, and will not affect the patient’s preoperative preparation.

In this study, there was no difference in the number of patients with adverse reactions at the site of electrical stimulation between TEAS group and control group. The electrical stimulation intensity in control group was only 5 mA, which was significantly lower than 13 mA in TEAS group. This may be explained by the redness, swelling, and itching of the stimulated parts on the skin of some patients being caused by the electrode adhesive of the stimulation device, not by the electrical stimulation itself.

We performed electrical stimulation in the control group in the trial mainly to make the participants blind to the grouping. Because the occurrence of preoperative hypertension is related to anxiety. If participants were not electrically stimulated and found themselves in the control group, it may increase the psychological load and affect the preoperative blood pressure, which in turn may affect the trial results. We artificially limited the electrical stimulation intensity of the control group. TEAS uses electrodes to act on a large area. The electrical stimulation at non-acupuncture points in the upper extremity may also affect the test results if the intensity of the stimulation is too high for the surrounding acupuncture points.

### 4.1 Limitations

This study has certain limitations. First, only the TEAS data of patients with gynecological malignant tumors were included. Therefore, the impact of TEAS on other types of patients undergoing different types of surgery—especially male patients—was not included and requires further study. Second, the duration of the influence of TEAS on blood pressure was not determined in this study. Further study is therefore needed to verify the duration of TEAS effects on blood pressure.

## 5 Conclusion

In summary, as a noninvasive method, TEAS was effective, economical, and safe. Transcutaneous electrical stimulation at the Neiguan and Hegu acupoints effectively regulated the preoperative blood pressure of gynecological malignant tumor patients with hypertension and reduced preinduction hypertension.

## Data Availability Statement

The raw data supporting the conclusions of this article will be made available by the authors, without undue reservation.

## Ethics Statement

The studies involving human participants were reviewed and approved by the Medical Ethics Committee of Shengjing Hospital of China Medical University. The patients/participants provided their written informed consent to participate in this study.

## Author Contributions

All authors contributed to the study conception and design. Material preparation and data collection were performed by LC, SL, and YC. Analysis was performed by YS and LC. The first draft of the manuscript was written by LC and YS, and all authors commented on previous versions of the manuscript. All authors read and approved the final manuscript.

## Conflict of Interest

The authors declare that the research was conducted in the absence of any commercial or financial relationships that could be construed as a potential conflict of interest.

## Publisher’s Note

All claims expressed in this article are solely those of the authors and do not necessarily represent those of their affiliated organizations, or those of the publisher, the editors and the reviewers. Any product that may be evaluated in this article, or claim that may be made by its manufacturer, is not guaranteed or endorsed by the publisher.
